# Blood purification therapy with a hemodiafilter featuring enhanced adsorptive properties for cytokine removal in patients presenting COVID-19: a pilot study

**DOI:** 10.1186/s13054-020-03322-6

**Published:** 2020-10-12

**Authors:** Gianluca Villa, Stefano Romagnoli, Silvia De Rosa, Massimiliano Greco, Marco Resta, Diego Pomarè Montin, Federico Prato, Francesco Patera, Fiorenza Ferrari, Giuseppe Rotondo, Claudio Ronco

**Affiliations:** 1 Department of Health Sciences, Section of Anaesthesiology, Intensive Care and Pain Medicine, University of Florence, Viale Pieraccini 6, 50139 Florence, Italy; 2 Department of Anaesthesia and Intensive Care, Azienda Ospedaliero Universitaria Careggi, Florence, Italy; 3 Department of Anesthesia and Intensive Care Unit, St. Bortolo Hospital, Vicenza, Italy; 4 International Renal Research Institute of Vicenza (IRRIV), Vicenza, Italy; 5 Department of Anesthesiology and Intensive Care, Humanitas Clinical and Research Center-IRCCS, Milano, Italy; 6 Department of Biomedical Sciences, Humanitas University, Milano, Italy; 7 Department of General Anesthesia and Intensive Care, IRCCS Policlinico San Donato, Milan, Italy; 8 Anesthesia and Intensive Care, Ospedale degli Infermi, Ponderano, Biella Italy; 9 Department of Nephrology, Dialysis and Transplantation Ospedale Santa Maria della Misericordia, Perugia, Italy; 10 Department of Anaesthesia and Intensive Care Unit, IRCCS San Matteo Hospital and University of Pavia, Pavia, Italy; 11 Maria Vittoria Hospital, Turin, Italy; 12 Department of Medicine, Università di Padova, Padova, Italy; 13 Department of Nephrology, Dialysis and Kidney Transplantation, San Bortolo Hospital, Vicenza, Italy

**Keywords:** Acute renal injury, IL-6, Multiorgan dysfunction, SOFA score

## Abstract

**Background:**

Systemic inflammation in COVID-19 often leads to multiple organ failure, including acute kidney injury (AKI). Renal replacement therapy (RRT) in combination with sequential extracorporeal blood purification therapies (EBP) might support renal function, attenuate systemic inflammation, and prevent or mitigate multiple organ dysfunctions in COVID-19.

**Aim:**

Describe overtime variations of clinical and biochemical features of critically ill patients with COVID-19 treated with EBP with a hemodiafilter characterized by enhanced cytokine adsorption properties.

**Methods:**

An observational prospective study assessing the outcome of patients with COVID-19 admitted to the ICU (February to April 2020) treated with EBP according to local practice. Main endpoints included overtime variation of IL-6 and multiorgan function-scores, mortality, and occurrence of technical complications or adverse events.

**Results:**

The study evaluated 37 patients. Median baseline IL-6 was 1230 pg/ml (IQR 895) and decreased overtime (*p* < 0.001 Kruskal-Wallis test) during the first 72 h of the treatment, with the most significant decrease in the first 24 h (*p* = 0.001). The reduction in serum IL-6 concentrations correlated with the improvement in organ function, as measured in the decrease of SOFA score (rho = 0.48, *p* = 0.0003). Median baseline SOFA was 13 (IQR 6) and decreased significantly overtime (*p* < 0.001 at Kruskal-Wallis test) during the first 72 h of the treatment, with the most significant decrease in the first 48 h (median 8 IQR 5, *p* = 0.001).

Compared to the expected mortality rates, as calculated by APACHE IV, the mean observed rates were 8.3% lower after treatment. The best improvement in mortality rate was observed in patients receiving EBP early on during the ICU stay. Premature clotting (running < 24 h) occurred in patients (18.9% of total) which featured higher effluent dose (median 33.6 ml/kg/h, IQR 9) and higher filtration fraction (median 31%, IQR 7.4). No electrolyte disorders, catheter displacement, circuit disconnection, unexpected bleeding, air, or thromboembolisms due to venous cannulation of EBP were recorded during the treatment. In one case, infection of vascular access occurred during RRT, requiring replacement.

**Conclusions:**

EBP with heparin-coated hemodiafilter featuring cytokine adsorption properties administered to patients with COVID-19 showed to be feasible and with no adverse events. During the treatment, patients experienced serum IL-6 level reduction, attenuation of systemic inflammation, multiorgan dysfunction improvement, and reduction in expected ICU mortality rate.

## Introduction

Kidney damage and acute kidney injury (AKI) appear to be a common finding among severely ill patients infected with severe acute respiratory syndrome coronavirus-2 (SARS-CoV-2) and affected by coronavirus disease 2019 (COVID-19). Up to 40% of all patients hospitalized with COVID-19 in China presented abnormal proteinuria upon admission, and between 20 and 40% of those admitted to the intensive care unit (ICU) experienced AKI [1–6]. Yet, these figures may underestimate kidney involvement [7], given the lack of information on patient baseline kidney function prior to hospital admission [8]. A recent study that investigated COVID-19 patients in the New York area showed that over 30% of the patients developed acute kidney injury (AKI). Among the 2634 hospitalized patients, 14.2% were admitted to intensive care unit (ICU). Patients who required mechanical ventilation developed AKI in 89.7% of the cases, compared to 21.7% of non-ventilated patients.

So far, AKI in critically ill patients with COVID-19 appears to be a marker of disease severity and a negative predictor of survival and is posing additional challenges to patient management [1,2;7]. Indeed, the exacerbated scenario observed in these patients appears to be compatible with the hyper-inflammatory state triggered by other coronavirus infections [9–11] and with the hypothesis of complex organ cross-talk in critical illness [12–14].

From a patient-management prospective, early reports on COVID-19 have documented the need for renal replacement therapy (RRT) in approximately 23–36% of patients [4;6] (vs 10% in the ICU general population [15]), ensuing after a median of 15 days after illness onset [1,2,5]. Also, 67% of these present additional multiple organ failure, suggesting a relevant role for organ support and cytokine blockade/removal [16]. Despite the efficacy of extracorporeal blood purification therapies (EBP) applied in hyper-inflammatory states have still not been established and their mechanisms are still the object of research, some authors have proposed RRT in combination with sequential EBP as a means to support renal function and attenuating systemic inflammation in COVID-19 [11].

Recently, Ronco et al. have addressed the peculiarities of kidney impairment in COVID-19 patients, highlighting the importance of abiding by the KDIGO guidelines and providing additional recommendations to comply with specific EBP, such as ways to deal with the typically observed hypercoagulation [11, 17]. Additional outcome benefits may also come with a pondered choice of specific technologically advanced EBP disposables, such as novel adsorbing cartridges, and the full exploitation of their distinguishing properties.

The oXiris membrane (Baxter, IL - USA) is a highly biocompatible heparin-coated hemodiafilter mostly known for its use in supporting renal function; however, the device can also be used for unselective removal of cytokines and endotoxin and features the added function of reducing clotting during treatment.

Hence, we aimed to preliminarily investigate the role of oXiris for the management of critically ill patients receiving EBP in the setting of COVID-19. In particular, we described the overtime variation of IL-6 [10, 18] and multiorgan function during EBP, and clinical severity scores as surrogate outcomes of feasibility in this patient population, for which there are still no published data.

## Methods

### Study design and patient population

The purpose of this work was to assess the feasibility of EBP treatment with oXiris in patients with COVID-19, by means of a prospective, multicenter, and observational study on data recorded into the oXirisNet Registry (aRRT - http://www.arrt.eu/) [19], an Italian registry on patients with multiple organ dysfunction who have undergone EBP with the oXiris membrane. The study considered data of all patients with a confirmed diagnosis of COVID-19 admitted to the ICU between February and April 2020, who received treatment with the oXiris membrane for immunomodulation and/or support to renal function during AKI.

Diagnosis of SARS-CoV-2 infection was defined as a positive outcome to a real-time reverse transcriptase-polymerase chain reaction (RT-PCR) at nasal/oral swab. AKI was defined in accordance with the KDIGO criteria. Previous treatments with other filters or further sequential treatment after treatment with oXiris represented criteria for exclusion from the study.

Finally, in order to describe the overtime variation of biochemical and clinical features among critically ill patients with SARS-CoV-2 infection but not treated with EBP, the study included data of a cohort of COVID-19 patients from the same centers.

### Study objectives, outcomes, and endpoints

Objectives of this preliminary study were (1) to assess the variation of IL-6 and multiorgan dysfunction scores overtime, (2) to compare observed versus predicted mortality rate in terms of disease severity scores, and (3) to assess the occurrence of procedure-related complications, clotting, and treatment duration.

### Measurements and parameters

Data considered for the study included main anthropometric, clinical and biochemical parameters (including inflammatory markers), comorbidities, disease-associated symptoms, and organ dysfunction severity indexes (APACHE IV score [20]; SOFA score). Measurement of IL-6 was evaluated by immunoassay analysis (Simple PlexTM, ProteinSimple, San Jose, California, USA) [21] across all centers. EBP details considered included data on prescription (e.g., flows, dose, and anticoagulation), delivery (e.g., filtration fraction or treatment effective time), and outcome (e.g., clotting and unintended discontinuation).

Clinical and technical evaluations were performed immediately before EBP initiation (T0), after 12 h (T1), and every 24 h thereafter for the first 10 days from T0. The follow-up ended either at ICU discharge or death if it occurred in the ICU.

### Instrumental and clinical interventions

All patients included in this prospective observational study received antimicrobials, EBP, mechanical ventilation, and any other supportive treatment in accordance with the clinical judgment of the treating center. Similarly, according to local practice, patients were placed in the prone position for at least 16 h a day when Pa = 2/FiO_2_ < 150 (moderate-severe ARDS) [22].

EBP was carried out with the oXiris hemodiafilter (all patients had been prospectively recorded into the oXirisNet Registry). The choice for the specific use of the oXiris membrane was based on a patient-basis personalized approach and clinical judgment of the ICU team in consideration of the membrane’s specific technical features. The membranes were used on the PrismaFlex systems (Baxter, IL - USA).

### Statistical analyses

The normality distribution of the variables was tested by the Chen-Shapiro test [23]. Descriptive statistics were performed. Frequencies and percentages were used for qualitative variables, while means and respective standard deviations were calculated for quantitative variables. Median and interquartile range (IQR) were calculated for quantitative variables with non-normal distribution.

Spearman’s and Pearson’s correlation coefficients were used to explore the relationship between quantitative variables. Kruskal-Wallis test was carried out to compare the groups for the variables with non-normal distribution. Sidak post hoc adjustments were performed for multiple comparisons.

In the regression analysis, the explanatory variables were coded as binary variables. For each of these events, we assessed by multivariate regression the combined effect of continuous and ordinal variables for which the previous univariate regression analysis had evidenced a significant effect at the time of the event. Boxplots were drawn to describe IL-6 and SOFA score variations.

No statistical comparisons were made between COVID-19 patients treated and not-treated with EBP, nor did we perform any sample calculation as this was a feasibility assessment and was åbeyond the scope of this study.

All calculations were carried out using a standard statistical package (STATA for Windows version 14.1, College Station, Texas, USA).

### Ethical concerns

The present study was approved by the local Ethics Committee, “Comitato Etico di Area Vasta Centro, Regione Toscana”, Florence, Italy [rif. CEAVC 14334]. Given its observational design, the study did not involve any medical, pharmacological, or behavioral interventions in addition to the standard practice implemented by the physicians regardless of the registry. All research has been carried forth in the agreement with the principles laid out in the original Declaration of Helsinki and its later amendments, and data were handled in agreement with patient informed consent.

## Results

The present assessment considered all patients from the oXirisNet Registry featuring a confirmed diagnosis of COVID-19, resulting in 37 (out of a total 95 patients in the registry), from four different hospitals. The most frequent COVID-19 symptoms since onset were cough in 25 patients (78%), fever in 30 (81%), dyspnea 29 (78.4%), and gastrointestinal manifestations in 2 patients (5.40%).

All patients included in the study required ICU admission for respiratory support. Four of these (10.8%) also required renal support, and 2 (5.4%) patients with shock required cardiovascular support. Extracorporeal treatment began after a median of 3.6 days (IQR 3.7) from ICU admission and 14 days (IQR 10) from symptom onset. Anthropometric parameters, comorbidities, and clinical conditions at the time of EBP initiation are described in Table 1.

**Table 1:** Patient anthropometric characteristics, comorbidities and clinical parameters at EBP initiation

Anthropometric data		Comorbidities	
Ethnicity		CKD	3 (8.1%)
Caucasian	35 (94.6%)	CLD (Child-Pugh ≥ B)	1 (2.7%)
Asian	2 (5.4%)	Diabetes	8 (21.6%)
Gender		Previous oncological disease	3 (8.1%)
Male	31 (83.8%)	Hypertension	11 (29.7%)
Female	6 (16.2%)	Cerebral vascular disease	1 (2.7%)
M/F	31/6	Coronary vascular disease	1 (2.7%)
Age (years)	59.5 ± 9.5	Systemic vascular disease	1 (2.7%)
Weight (Kg)	90 ± 19	Chronic steroid tt	1 (2.7%)
Height (cm)	171 ± 8	Obesity	20 (54.1%)
BMI	30.3 (5.7)	Other	13 (35.1%)
**Clinical data at RRT initiation**			
GCS		Lactates (mmol/L)	1.67 (1.2)
3	36 (97.3%)	Sodium (mmol/L)	138 (10)
4	1 (2.7%)	Potassium (mmol/L)	4.4 ± 0.7
Heart rate (bpm)	91 ± 21	Magnesium (mg/dL)	2.3 ± 0.7
Rhythm		Phosphate (mg/dL)	4.7 ± 1.8
Rhythmic	31 (83.8%)	Bicarbonate (mEq/L)	23 ± 5
Arrhythmic	6 (16.2%)	Hematocrit (%)	33.8 ± 7.4
Systolic pressure (mmHg)	120 (30)	Baseline creatinine (mg/dL)	0.89 (0.2)
Diastolic pressure (mmHg)	63 ± 12	Current creatinine (mg/dL)	2.26 (2.5)
Mean pressure (mmHg)	84 (23)	Urinary output (ml/h)	30 (40)
Vasoactive	24 (64.9%)	24 h urinary output (ml)	1000 (1250)
Adrenaline	2 (5.4%), 0.03 ± 0.01 μg/kg/min	Urea (mg/dL)	71 (139)
Noradrenaline	24 (64.9%), 0.20 ± 0.19 μg/kg/min	Bilirubin (mg/dl)	0.8 (2.5)
Vasopressin	1 (2.7%), 1.6 U/h	Albumin (g/dl)	2.64 (0.42)
Dobutamine	1 (2.7%), 8 μg/kg/min	Platelets (10^3/μl)	231 (177)
Dopamine	1 (2.7%), 3 μg/kg/min	INR	1.1 (0.3)
Vasoactive Inotropic Score (VIS)	10 (22)	Antithrombin (%)	71 ± 26
V_T_ settings (ml)	459 ± 63	Fibrinogen (mg/dl)	640 (229)
RR (breaths/min)	23 ± 4	D-dimers (ng/ml)	3240 (8417)
PEEP (cmH_2_O)	13 ± 3	PCT (ng/ml)	0.78 (2.59)
PIP (cmH_2_O)	30 (9)	Temp (°C)	36.7 ± 0.9
Mean airway pressure (cmH_2_O)	30	CRP (mg/L)	200 (176)
P_Plat_ (cmH_2_O)	26 ± 3	WBC (10^3/μl)	10.47 (6.73)
Compliance	39 ± 12	Ferritin (ng/ml)	12.3 ± 5.9
FiO_2_ (%)	70 (19)	IL-6 (ng/l)	1230 (895)
PaO_2_	83 ± 23	CD 4+	164.9 ± 122.5
PaO_2_/FiO_2_	119 ± 42	Crystalloid prev.12 h (ml)	906 ± 693
SaO_2_ (%)	95 (3)	APACHE IV	117 ± 20
PaCO_2_ (mmHg)	51 ± 11	Predicted mortality (%)	64.7 ± 16.2
A-a O_2_ gradient	377.7 ± 113.1	Total SOFA Score	13 (6)
pH	7.31 ± 0.10		

*CKD* chronic kidney disease, *CLD* chronic liver disease, *CRP* C-reactive protein, *INR* international normalized ratio, *MV* mechanical ventilation, *OTI* oro-tracheal intubation, *PCT* procalcitonin, *PIP* peak inspiratory pressure, *P*_*plat*_ plateau pressure, *RR* respiratory rate, *V*_*T*_ tidal volume, *WBC* white blood cells

Indications for EBP with oXiris in this cohort were biochemical and clinical evidence of systemic inflammation associated with (1) AKI with absolute indications for RRT or (2) hemodynamic instability and/or multiorgan dysfunction in patients whose renal functional reserve was considered not adequate to sustain the metabolic burden of expected fluid overload. The median treatment time was 37 h (IQR 56).

AKI was the cause of initiation of EBP in 26 (70.3%) patients; 20 of them presented a KDIGO stage 3, while 6 a KDIGO stage 2. A specific indication of treatment for these patients was fluid overload in 15 (40.5%) patients, control of uremic solutes in 18 (48.6%), and adjustment of hydroelectrolytic balance in 9 (24.3%) patients. The remaining 11 (29.7%) patients were treated with EBP even in the absence of renal indications; all of them were in KDIGO stage 1. Upon EBP initiation, all patients were on controlled invasive mechanical ventilation; 24 (64.9%) of these also presented hemodynamic instability, which was treated with vasoactive amines. Fifteen patients were under diuretic treatment—all with furosemide (500 mg/day). A pre-hospital admission value of serum creatinine was available for 14 (37.8%) of the 37 patients included in the study.

The initial prescription was continuous veno-venous hemodiafiltration (CVVHDF) in all cases. Treatment settings are summarized in Table 2.

**Table 2:** EBP initial prescription

Venous cannulation	
*Right internal jugular*	6 (16.2%)
*Right subclavian*	1 (2.7%)
*Right femoral*	26 (70.3%)
*Left femoral*	4 (10.8%)
**Initial prescription to treatment**	
*Qb (ml/min)*	150 (0)
*Qd (ml/h)*	1000 (600)
*Qr pre-dilution (ml/h)*	0 (0)
*Qr post dilution (ml/h)*	726 ± 510
*Q PBP - citrate infusion (ml/h)*	1400 (1000)
*UF net (ml/h)*	50 (100)
*Current dose (ml/kg/hr)*	31 (11)
*Filtration fraction (%)*	28.3 ± 6.8

*Qb* blood flow, *Qd*, dialysate flow, *Qr pre* replacement flow in predilution, *Qr post* replacement flow in postdilution, *Q PBP* citrate flow infused by pre-blood pump, *UN net* net ultrafiltration

Among these, 4 treatments were performed without anticoagulation; 6 were performed with systemic anticoagulation with heparin, with initial bolus given to only 3 patients (average dose 35 U/kg). The average heparin infusion for these 6 patients was 10 U/kg/h. Finally, 27 patients were treated with regional anticoagulation with citrate-calcium; the citrate (trisodium citrate 18/0) dose was 3 mmol/L with a 100% calcium chloride replacement.

### IL-6 and SOFA score over time variation

The median baseline IL-6 was 1230 pg/ml (IQR 895) and correlated with the severity of baseline SOFA score (rho = 0.44, *p* = 0.03) and in particular with the PaO_2_/FiO_2_ ratio (rho = − 0.47, *p* = 0.02).

**Fig. 1:**
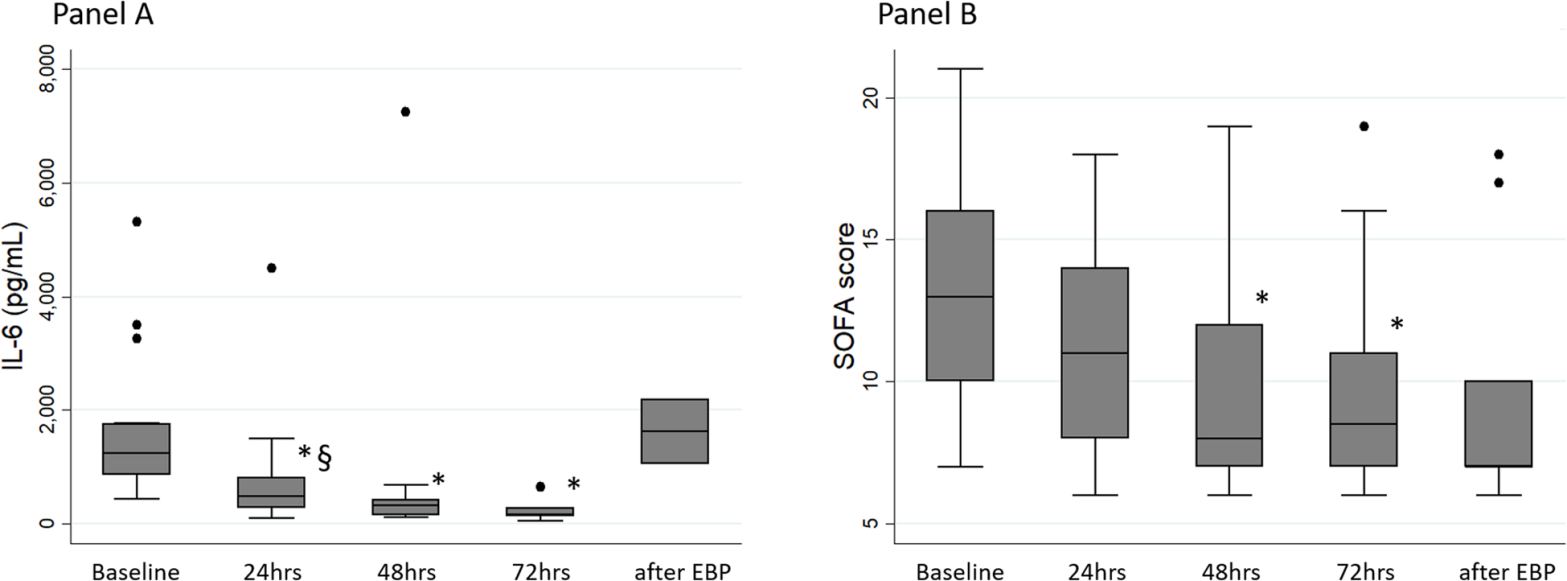
IL-6 and SOFA score variations overtime. IL-6 (**a**) and SOFA score (**b**) at baseline, at 24 h, 48 h, and 72 h of treatment initiation and immediately after the EBP discontinuation for the entire population. *Statistical significance with respect to the baseline (*p* < 0.016); §Statistical significance with respect to the previous time point (*p* < 0.016)

During the first 72 h of the treatment, IL-6 significantly decreased overtime (*p* < 0.001 at Kruskal-Wallis test) (see Fig. 1a). In particular, the IL-6 serum concentration fell to 479 pg/ml (IQR 531) at 24 h to 320 pg/ml (IQR 259) at 48 h and to 160 pg/ml (IQR 141) at 72 h (*p* = 0.001 for each time point if compared to the baseline; a *p* = 0.05/3 = 0.016 was considered for statistical significance). Serum IL-6 reduction was statistically significant in the first 24 h of treatment (*p* = 0.001), but not in the second 24 h (∆ IL-6 = 197.5 pg/ml, *p* = 0.17, IQR 323) or third 24 h (∆ IL-6 = 118 pg/ml, IQR 100.5, *p* = 0.16) (a *p* = 0.05/3 = 0.016 was considered for statistical significance) (see Fig. 1a). Increased values of serum IL-6 were observed immediately after the EBP discontinuation (median 1615 pg/ml, IQR 1149).

The reduction in serum IL-6 concentrations correlated with improvement in organ function, as measured in a decrease of SOFA score (rho = 0.48, *p* = 0.0003). Such reduction observed during the first 72 h of treatment was statistically significant (*p* = 0.002 at Kruskal-Wallis test) (see Fig. 1b). Median values of SOFA score at main time points were 13 (IQR 6) at the baseline, 11 (IQR 6) at 24 h (*p* = 0.09), 8 (IQR 5) at 48 h (*p* = 0.001), 8.5 (IQR 4) at 72 h (*p* = 0.002); a *p* = 0.05/3 = 0.016 was considered for statistical significance (see Fig. 1b). The median SOFA score immediately after the EBP discontinuation was 7 (IQR 0).

With specific reference to organ functional recovery and mortality, the SOFA items that showed the most improvement with treatment related to the hemodynamic stability (in terms of Vasoactive Inotropic Score and vasoactive requirements in the first 72 h) and lung functions (quantified in terms of PaO_2_/FiO_2_ ratio).

The variation of the clinical and prescription parameters of the EBP at the various time points is described in Table 3.

**Table 3:** Main clinical parameters at monitoring time points 12, 24, 48, and 72 h

	12 h (***n*** = 31)	24 h (***n*** = 30)	48 h (***n*** = 27)	72 h (***n*** = 22)
**Clinical parameteres upon RRT initiation**				
GCS	3 (31 100%)	3 (30 100%)	3 (25 92.6%)	3 (20 90.9%)
			7 (1 3.7%)	6 (1 4.5%)
			8 (1 3.7%)	9 (1 4.5%)
Heart rate (bpm)	87.82 ± 18.42	93.62 ± 21.76	90.70 ± 17.45	90.73 ± 17.63
Rhythm				
Rhythmic	26 (83.9%)	25 (83.3%)	25 (92.6%)	20 (90.9%)
Arrhythmic	4 (12.9%)	5 (16.7%)	2 (7.4%)	2 (9.1%)
Systolic pressure (mmHg)	118 (19)	120 (30)	120 (33)	132 (40)
Diastolic pressure (mmHg)	64 ± 10	67 ± 10	66 ± 12	67 ± 14
Mean pressure (mmHg)	80 (13)	84 (17)	87 (24)	88 (26)
Vasoactive drugs	19 (51.4%)	11 (36.7%)	5 (18.5%)	5 (22.7%)
Adrenaline	0 (0%)	0 (0%)	0 (0%)	0 (0%)
Noradrenaline	19 (51.4%)	11 (36.7%)	5 (18.5%)	5 (22.7%)
Vasopressin	0 (0%)	0 (0%)	0 (0%)	0 (0%)
Dobutamine	1 (3.2%)	1 (3.3%)	0 (0%)	0 (0%)
Dopamine	0 (0%)	0 (0%)	0 (0%)	0 (0%)
Vasoactive Inotropic Score (VIS)	4 (10)	0 (12.5)	0 (0)	0 (0)
V_T_ settings (ml)	427 ± 73	433 ± 72	484 ± 71	467 ± 69
RR (breaths/min)	23 ± 4	22 ± 5	23 ± 5	24 ± 4
PEEP (cmH_2_O)	13 ± 3	12 ± 3	12 ± 3	12 ± 3
PIP (cmH_2_O)	312 ± 8	30 ± 8	29 ± 13	40 ± 9
Mean airway pressure (cmH_2_O)	25 (6)	24 (3)	23 (3)	23 (3)
P_Plat_ (cmH_2_O)	26 ± 4	25 ± 4	26 ± 5	27 ± 5
Compliance	39 ± 10	44 ± 16	39 ± 13	38 ± 14
FiO_2_ (%)	70 (20)	67.5 (25)	60 (20)	60 (20)
PaO_2_ (mmHg)	96 ± 30	91 ± 27	101 ± 30	98 ± 28
PaO_2_/FiO_2_	145 ± 56	146 ± 55	170 ± 57	168 ± 47
SaO_2_ (%)	96 (4)	96 (4)	97 (3)	96 (4)
PaCO_2_ (mmHg)	50 ± 9	50 ± 11	51 ± 13	54 ± 16
A-a O_2_ gradient	339.1 ± 112.8	318.2 ± 113.3	280.8 ± 108.7	263.8 ± 79.9
pH	7.32 ± 0.08	7.36 ± 0.08	7.39 ± 0.07	7.39 ± 0.04
Lactates (mmol/L)	14 (10.2)	1.4 (0.9)	1.2 (0.4)	1.1 (0.4)
Sodium (mmol/L)	139 (6)	138 (5)	136 (4)	135 (7)
Potassium (mmol/L)	4.1 ± 0.7	4.1 ± 0.7	4.1 ± 0.6	4 ± 0.6
Magnesium (mg/dL)	2 ± 0.6	2 ± 0.6	1.7 ± 0.5	1.8 ± 0.4
Phosphate (mg/dL)	3.3 ± 1.2	3.6 ± 1.1	3.4 ± 1.2	3.5 ± 1
Bicarbonate (mEq/L)	24 ± 4	23 ± 3	24 ± 3	24 ± 3
Hematocrit (%)	34 ± 5.2	33.3 ± 6.6	33.3 ± 6.4	32.8 ± 5.3
Current creatinine (mg/dL)	1.92 (1.31)	1.34 (1.19)	1.31 (0.85)	1.36 (1.31)
Urinary output (ml/h)	25 (45)	15 (65)	10 (50)	2.5 (80)
24 h urinary output	400 (855)	510 (1245)	120 (1470)	90 (1485)
Urea (mg/dL)	54 (92)	51 (60)	47.5 (33)	48 (39)
Bilirubin (mg/dl)	0.8 (1.7)	0.8 (1.9)	0.7 (1.6)	0.7 (1.6)
Albumin (g/dl)	2.7 (0.3)	2.8 (0.3)	2.8 (0.4)	2.9 (0.4)
Platelets (10^3/μl)	210 (145)	219 (150)	215 (148)	218 (115)
INR	1.1 (0.3)	1.1 (0.3)	1.1 (0.1)	1.1 (0.1)
Antithrombin (%)	76 ± 26	83 ± 24	84 ± 19	84 ± 13
Fibrinogen (mg/dl)	650 (163)	611 (183)	630 (323)	624 (250)
D-dimers (ng/ml)	2900 (5845)	2500 (5608)	1629 (3513)	1500 (1900)
PCT (ng/ml)	0.62 (2.07)	0.62 (2.07)	0.56 (2.15)	1.1 (1.36)
Temperature (°C)	36.2 ± 0.7	36.2 ± 0.9	36.4 ± 0.8	36 ± 0.7
CRP (mg/L)	150 (123.2)	135 (131)	133 (130)	128 (122)
WBC (10^3/μl)	11 (5.87)	10.90 (6.62)	12.55 (8.43)	14.32 (6.75)
Ferritin (ng/ml)	408 ± 333	900 ± 887	761 ± 1045	2033 ± 2855
IL-6 (ng/l)	631.5 (165)	645 (881)	320 (259)	159.5 (141)
Total SOFA Score	12 (7)	11 (6.5)	8 (5)	8.5 (4)
**Treatment prescription**				
Qb (ml/min)	150 (20)	150 (20)	150 (20)	150 (0)
Qd (ml/h)	1000 (700)	1000 (600)	1000 (700)	1000 (600)
Qr pre dilution (ml/h)	0 (0)	0 (0)	0 (0)	0 (0)
Qr post dilution (ml/h)	630 ± 414	665 ± 446	527 ± 347	606 ± 320
Q PBP - citrate infusion (ml/h)	1400 (500)	1350 (500)	1500 (300)	1500 (200)
UF net (ml/h)	80 (100)	70 (100)	50 (100)	100 (130)
Current dose (ml/kg/h)	29 (8)	29 (10)	29 (7)	30 (8)
Filtration fraction (%)	28.5 ± 5.3	27.9 ± 7	28.2 ± 6	28.2 ± 6.8

Data are expressed as percentage *N* (%), mean ± standard deviation (mean ± SD), and median (interquartile range, IQR) as appropriate. *CKD* chronic kidney disease, *CLD* chronic liver disease, *CRP* C-reactive protein, *INR* international normalized ratio, *MV* mechanical ventilation, *OTI* oro-tracheal intubation, *PCT* procalcitonin, *PIP* peak inspiratory pressure, *P*_*plat*_ plateau pressure, *RASS* Richmond Agitation-Sedation Score, *RR* respiratory rate, *V*_*T*_ tidal volume, *WBC* white blood cells

Finally, eight COVID-19 patients not treated with EBT were also retrospectively observed as to overtime variation of the clinical and biochemical parameters (including the natural decay of IL-6 and SOFA score variation) (See Supplementary material Table S1).

### Pharmacological treatment

In this cohort, every patient was treated with hydroxychloroquine and azitromycine, while no patient was treated with corticosteroids. Among them, 15 (40.5%) were also treated with tocilizumab, and 9 (24.3%) with lopinavir-ritonavir. Baseline levels and overtime variations in IL-6 and SOFA score in patients treated with tocilizumab and/or lopinavir-ritornavir are described in Table 4.

**Table 4:** Baseline levels and overtime variations in IL-6 and SOFA score in patients treated with tocilizumab and/or lopinavir-ritornavir

		***n*** (%)	Baseline SOFA score	Baseline IL-6	Δ SOFA score at 24 h	Δ IL-6 at 24 h	Mortality rate
Tocilizumab		15 (40.5%)	13 (IQR 3.5)	1598 (IQR 1635)*	− 1 (IQR − 2.3)	− 642 (IQR − 548)	8 (53.3%)
	Lop_Rit	2 (5.4%)	13 (IQR 2)	1125 (IQR 275)	− 4.00 (IQR 0)§	− 571 (IQR −71)	1 (50.0%)
	No-Lop_Rit	13 (35.1%)	13 (IQR 3)	1753 (IQR 1933)	− 0.50 (IQR − 1)§	− 904 (IQR − 631)	7 (53.8%)
No-Tocilizumab		22 (59.5%)	13 (IQR 7.8)	1089 (IQR 67)*	0 (IQR − 1)	− 552 (IQR − 526)	13 (59.1%)
	Lop_Rit	7 (18.9%)	14 (IQR 5.5)	1180 (IQR 80)	− 1 (IQR − 2)	− 702 (IQR − 15)	3 (42.9%)
	No-Lop_Rit	15 (40.5%)	13 (IQR 8.5)	794 (IQR 607)	0 (IQR − 1)	− 401 (IQR − 601)	10 (66.7%)

(Lop_Rit). *p = 0.02, §*p* = 0.049

### Predicted vs observed mortality rate

The expected mortality rate calculated based on the baseline APACHE IV score (117 ± 20) was 64.7% ± 16.2, whereas the ICU mortality observed was 56.4%, with a 8.3% mean difference. Stratifying patients based on ICU death, deceased patients had a longer time-to-EBP from the onset of COVID-19 symptoms compared to non-deceased patients (*p* < 0.001), independently from baseline SOFA score (*p* = 0.478 at multivariate logistic regression analysis adjusted for SOFA score).

The difference between expected and observed mortality was further analyzed by discriminating between early and delayed treatment first in terms of time and then of treatment needs. In the former case, patients were divided into early and delayed using cut-off as the median time-to-EBP of 14 days, which evidenced a 47.4% mortality rate for patients receiving early treatment against the 73.3% (IQR 34.3) expected mortality rate (median difference was 25.9%, IQR 33.6). Patients receiving delayed treatment had a 66.6% mortality rate compared to the 67.2% (IQR 11.3) expected APACHE IV mortality rate; (median difference was 0.1% (IQR 10.2).

Similarly, a greater decreasing trend was seen when patients were divided into early and delayed treatment by immunomodulation alone versus immunomodulation and renal support; patients who received EBP for immunomodulation alone had a 16.5% (IQR 18) reduction in observed mortality (compared to predicted) against a 1.1% (IQR 25.5) reduction for patients treated for both immunomodulation and renal support. Baseline levels of IL-6 and SOFA score were similar between patients with early and delayed treatments (*p* > 0.05 for both).

### Technical complications or adverse events

Table 5 describes the treatment trend over time: 7 treatments (18.9%) prematurely clotted (running < 24 h); 2 of them were carried out without anticoagulation (50% of all treatments performed without anticoagulation), while 5 with regional citrate anticoagulation (18.6% of all treatment performed with citrate). These 7 treatments had also higher effluent dose (median 33.6 ml/kg/hr., IQR 9) and higher filtration fraction (median 31%, IQR 7.4). No electrolyte disorders were recorded during the treatment, nor catheter displacement, circuit disconnection, unexpected bleeding, air, or thromboembolisms due to venous cannulation of EBP performance. As to adverse events/complications, only one occurred related to infection of the vascular access during the extracorporeal treatment, which required replacement of the access.

**Table 5:** Treatment trend overtime according to the prescribed anticoagulation

			T12h	T24h	T48h	T72h
No-Anticoagulation (*n* = 4)	Still running at that time point		2 (50%)	0		
	Withdrawn before that time point		2 (50%)	2 (50%)		
		Patient death	1 (50%)			
		Circuit clotting	1 (50%)	1 (50%)		
		Voluntary interruption		1 (50%)		
		*Max circuit life (72hrs)*	*0 (0%)*			
Heparin (*n* = 6)	Still running at that time point		4 (66.7%)	3 (50%)	2 (33.3%)	1 (16.7%)
	Withdrawn before that time point		2 (33.3%))	1 (16.7%)	2 (33.3%)	
		Patient death	1 (50%)	1 (100%)		
		Circuit clotting				
		Voluntary interruption	1(50%)		2 (100%)	
		*Max circuit life (72hrs)*	*1 (16.7%)*			
Citrate (*n* = 27)	Still running at that time point		22 (81.5%)	17 (63%)	13 (48.1 %)	6 (22.2%)
	Withdrawn before that time point		5 (18.5%)	5 (18.5%)	4 (14.8%)	7 (25.9%)
		Patient death	2 (40%)	1 (20%)	1 (25%)	3 (42.9%)
		Circuit clotting	2 (40%)	3 (60%)	1 (25%)	
		Voluntary interruption	1 (20%)	1 (20%)	2 (50%)	4 (57.1%)
		*Max circuit life (72hrs)*	*6 (22.2%)*			

## Discussion

The present feasibility assessment aimed to preliminarily investigate clinical and laboratory data in critically ill ICU patients with COVID-19, undergoing EBP with highly biocompatible membranes characterized by enhanced adsorptive properties for the cytokine. Levels of IL-6 markedly decreased in the first 24 h of treatment. This result was mirrored by improved SOFA scores, particularly for hemodynamic stability and pulmonary function. A slight decrease in observed vs predicted mortality rates as predicted by APACHE IV score was also observed. Early treatment yielded the best outcomes.

The oXiris membrane is a hemodiafilter pregrafted with an average of 4.500 UI/m^2^ heparin during manufacturing, with a surface polyethyleneimine (PEI) treatment providing a high amount of free positively charged amino groups that allows to adsorb large weight molecules such as endotoxin [9]. Previous studies have addressed the use of this membrane in critically ill patients with AKI [9], with a few specifically assessing its safety and feasibility in septic patients [9, 24–26]. So far, the data presented herein are the first published data on patients with COVID-19.

The study by Turani et al. [23] evaluated the use of oXiris in approximately 60 patients and confirmed the decrease in cytokine and endotoxin levels, as well as improved mean SOFA scores (from 12.4 to 9, with 72 ± 13 h treatments), cardiorenal function and respiratory parameters with a decreased of noradrenaline dosage. A case report series by Zhang et al. [24] on four septic ICU patients with AKI supports the use of the membrane for early treatment as an adjunctive therapy, provided there be in place a proper infection control. Importantly, the authors also documented the outcome on patients with a high risk of bleeding, reporting the feasibility in these patients, without additional anticoagulation for up to 36 h. Finally, a randomized control study by Broman et al. [25] on a small sample of septic patients with AKI, comparing oXiris with a standard membrane, found decreased endotoxin levels on approximately a third of the patients, and lower TNF-alpha, IL-6, and INF gamma compared to the standard. Reductions were significant in the first treatment period. Moreover, norepinephrine administration decreased with oXiris but not with the standard filter.

### Cytokines, IL-6, and organ dysfunction

With a specific reference to the inflammatory response, results from our population confirm the correlation between serum concentration of pro-inflammatory cytokines (such as IL-6) and multiorgan dysfunction. IL-6 is a leading mediator influencing systemic inflammation and has shown increased concentrations among COVID-19 patients with ARDS [27]. Higher concentrations of cytokines in COVID-19 patients are associated with organ dysfunction and worse outcome, and generally, the higher IL-6 in the blood, the higher level of SOFA score [13]. COVID-19 patients considered by the physician as “not severely inflamed” and who therefore did not undergo EBP (Table S1) had lower levels of IL-6 and SOFA score than those undergoing EBP with the oXiris membrane. Application of EBP, in this context, has been suggested by some authors for cytokine removal [14, 28] and thus as prevention or attenuation of inflammatory-related organ damage. In this context, the oXiris membrane has been proven to remove cytokines and endotoxin via unselective adsorption. Interestingly, a net decrease in IL-6 was observed in this study during EBP, along with a stable reduction of SOFA score, independently from other pharmacological therapies such as tocilizumab [27]. In accordance with other observational findings in critically ill septic patients, also in this study, the improvement in multiorgan dysfunction during EBP was mainly related to the restoring of hemodynamic stability (expressed in terms of vasoactive inotropic score) and oxygenation (measured as PaO_2_/FiO_2_). Although these reductions cannot be univocally advocated to EBP with oXiris in this study, our results seem to suggest a potential role of this treatment in reducing inflammatory mediators and improving multiorgan function. Table S1 shows the natural decay of IL-6 in critically ill patients with SARS-CoV-2 infection not treated with EBP admitted to the same enrolling centers. Clinical comparison with the overtime variation of IL-6 observed in COVID-19 patients treated with oXiris is particularly interesting. In particular, in the latter cohort, IL-6 serum concentrations markedly fell within the first 24 h of extracorporeal treatment, reaching asymptotically a steady state. IL-6 reduction was statistically significant in the first 24 h of treatment, becoming less important through the following 48 h and 72 h. Technically, this phenomenon might be explained considering the expected membrane fouling that occurs during continuous extracorporeal therapies (running for 24–72 h) and the expected reduction of adsorption properties due to overtime saturation of sites available for the unselective link between solutes and the membrane. For these reasons, a scheduled replacement of the membrane every 24 h might be considered in order to maximize the clearance effect over time [11]. In fact, this aspect should be carefully considered in a pandemic period that calls for careful allocation of limited resources available.

Interestingly, a slight increase in serum IL-6 can be observed at the EBP discontinuation. This effect may further suggest that EBP might have had an effect on maintaining serum IL-6 concentrations stable and low during the treatment. Nevertheless, this “rebound” in circulating inflammatory mediators (likely due to the increased IL-6 half-life related to the tocilizumab infusion [29, 30]) seems uncorrelated with the worsening of multiorgan function and naturally resolves in 2–4 days.

Notably, results on IL-6 and SOFA score overtime reduction should be carefully interpreted, considering that data might be positively influenced by the rate of drop-off of patients who died within the 72 h of observation (being those with worst values).

### Decreased mortality

Although the pathophysiological rationale of the use of EBP is to attenuate systemic inflammation and prevent or mitigate multiple organ dysfunction as short-time outcomes, in this study, we also observed a slight though an interesting decrease in observed mortality vs predicted mortality rates predicted by APACHE IV score. Compared to the 64.7% expected mortality for our population, calculated based on the APACHE IV score, the observed rate markedly decreased after treatment down to 56% (in contrast with the 80% mortality reported for COVID-19 patients admitted in the ICU in China) [3, 5]. The comparison between expected and observed mortality rate was used as a surrogate outcome to better describe the potential role of EBP in critically ill patients with systemic inflammation. Interestingly, reductions in mortality rate were observed for all patients independently from baseline SOFA score and were most marked in those who underwent an early EBP both in terms of timing (time from COVID-19 symptoms and EBP initiation) and indication for extracorporeal treatment (proactive treatment for immunomodulation prior to the need of renal support due to AKI). In general, the time from symptom onset to extracorporeal therapy initiation in our population was in agreement with data from recent studies of COVID-19 patients [1–3], with a median time of 14 days from symptom onset (vs 15 days in previous studies) [2, 4].

### Technical complications or adverse events

Overall, the treatment was administered with no specific complications, such as catheter displacement, accidental disconnection, bleeding, thromboembolisms, air embolism, or electrolyte disorders. In particular, changing the patient to a prone position did not affect the feasibility of EBP, which continued according to prescription. Such a result is extremely positive and addresses a shared concern among anesthesiologists on complications during maneuvers turning the patient with a large venous access. Despite many publications often describe extracorporeal therapies in COVID-19 as unfeasible due to hypercoagulability leading to unintended discontinuation of the treatment [29], clotting rates in our patients were similar to continuous RRT performed on other critically ill patients. Certainly, the delivery of extracorporeal therapies requires a more careful management of pharmacological and non-pharmacological strategies to reduce the occurrence of clotting. Some specific examples are the proper positioning a large vascular access—as recently recommended by an expert panel [11]—which has proven to be very effective in providing the adequate blood flow reducing filtration fraction or the optimization of anticoagulation (either with systemic heparinization or regional citrate). In our study, most patients underwent femoral venous cannulation with a vascular access over 25 cm, which guaranteed an optimal performance in terms of the blood flow (never below 150 ml/min). Similar to what was reported in other papers on treatment duration, treatments performed with regional citrate anticoagulation in our study also had a longer duration and were less affected by unintentional early interruption due to clotting. Interestingly, each RRT that had prematurely clotted (treatment < 24 h) had higher effluent doses (often prescribed to compensate the expected downtime of these patients) and higher filtration fraction (> 30%). However, beside these “classical approaches,” other newly developed concepts and technological advances that reduce membrane fouling and preserve extracorporeal clearance in patients with COVID-19 might also be considered. For instance, the use of highly biocompatible heparin-coated disposables such as the oXiris membrane might prevent local clotting activation and further prevent membrane fouling independently of the anticoagulation strategy applied.

### Strengths and limitations

The present study is the first to provide data on the use of this heparin-coated hemodiafilter in severely ill patients with SARS-CoV-2 infection, and it is potentially of use for conditions of hypercoagulation and hyperinflammation in COVID-19.

Given the observational nature of this study, we were unable to provide evidence of a causal relationship between this specific EBP and better patient outcomes nor establish the efficacy of EBP in improving organ dysfunction and ICU mortality. This would require further controlled studies. Also, given the few patients included, results did not point to any values or illness stage (with or with/without AKI) that might indicate a threshold or cutoff for the best moment for EBP initiation.

One aspect that was not addressed was an effluent drug and cytokine removal. Antibiotics and vasopressors are theoretically cleared by high-flux hemodiafilters (as the oXiris membrane is). Nevertheless, extracorporeal clearance of drugs specifically used for COVID-19 is theoretically not expected because of the drug’s molecular mass, surface electric charges, and protein binding. Tocilizumab, in particular, is unlikely removed by high-flux hemodiafilters considering its high molecular weight (149 KDa) [31]; furthermore, the electric charge of this complex protein likely reduces interactions with the filter membrane for the Gibbs-Donnan effects. Cytokines were not assessed nor in the effluent (to demonstrate the transmembrane clearance of this solutes across the high-flux membrane of oXiris), neither pre- and post-filter (to assess the adsorption of these solutes into the membrane). The overtime reduction of IL-6 during the treatments cannot thus be formally attributed to the EBP effect. Endotoxin removal was also not addressed due to the lack of specific measurements, but this was beyond the scope of our study.

## Conclusions

Critically ill patients with COVID-19 often present systemic inflammation and organ dysfunction requiring immunomodulation and RRT. EBP has been demonstrated to immunomodulate patients with maladaptive inflammatory response. A strong physiopatologic rationale thus supports the use of EBP in COVID-19. In particular, EBP may attenuate systemic inflammation, preventing or mitigating multiple organ dysfunction. In our population, all patients showed significant IL-6 reduction and an associated improvement in multiorgan dysfunction, particularly for short-term outcomes such as hemodynamic stability and oxygenation index. Moreover, EBP with the oXiris membrane resulted technically feasible and not associated with major adverse events. Nevertheless, future randomized trials are certainly required to demonstrate the clinical effects of EBP in COVID-19.

## Supplementary information

**Additional file 1: Table S1.** Main clinical parameters at monitoring time points 12, 24, 48 and 72 hours for control group. No patient had signs of severe systemic inflammation; only one had AKI (KDIGO stage 1); all of them had acute respiratory failure and were treated with invasive mechanical ventilation at the ICU admission. In this cohort, every patient was treated with hydroxychloroquine and azitromycine; two of them underwent treatement with lopinavir/ritornavir, while none with tocilizumab. Data are expressed as percentage N (%), mean ± standard deviation (mean±SD) and median (interquartile range, IQR) as appropriate. CKD: chronic kidney disease; CLD: chronic liver disease; CRP: C-reactive protein; INR: International Normalized Ratio; MV: mechanical ventilation; OTI: Oro-Tracheal intubation; PCT: procalcitonin; PIP: Peak inspiratory pressure; P_plat_: Plateau pressure; RASS: Richmond Agitation-Sedation Score; RR: Respiratory rate; V_T_: tidal volume; WBC: white blood cells.

## Data Availability

Data used for this study are available on the oXirisNet Registry (aRRT - http://www.arrt.eu/).

## Abbreviations

AKI: Acute kidney injury
APACHE: Acute Physiologic Assessment and Chronic Health Evaluation
CVVHDF: Continuous veno-venous hemodiafiltration
EBP: Extracorporeal blood purification
RRT: Renal replacement therapy
SOFA: Sequential Organ Failure Assessment

## References

[1] Zhou F, Yu T, Du R, Fan G, Liu Y, Liu Z (2020). Clinical course and risk factors for mortality of adult inpatients with COVID-19 in Wuhan, China: a retrospective cohort study. Lancet.

[2] Chen N, Zhou M, Dong X, Qu J, Gong F, Han Y (2020). Epidemiological and clinical characteristics of 99 cases of 2019 novel coronavirus pneumonia in Wuhan, China: a descriptive study. Lancet.

[3] Yang X, Yu Y, Xu J, Shu H, Xia J, Liu H, et al. Clinical course and outcomes of critically ill patients with SARS-CoV-2 pneumonia in Wuhan, China: a single-centered, retrospective, observational study. Lancet Respir Med 2020; Lancet Respir Med 2020. 10.1016/S2213-2600(20)30079-5. 10.1016/S2213-2600(20)30079-5 PMC7102538 32105632

[4] Huang C, Wang Y, Li X, Ren L, Zhao J, Hu Y (2020). Clinical features of patients infected with 2019 novel coronavirus in Wuhan, China. Lancet.

[5] Richardson S, Hirsch JS, Narasimhan M, Crawford JM, McGinn T, Davidson KW, et al. Presenting characteristics, comorbidities, and outcomes among 5700 patients hospitalized with COVID-19 in the New York City area. JAMA. 2020. 10.1001/jama.2020.6775. 10.1001/jama.2020.6775 PMC7177629 32320003

[6] Hirsch J, Ng J, Ross D, Sharma P, Shah H, Barnett R, et al. Acute kidney injury in patients hospitalized with COVID-19. Kidney Int. 2020; 10.1016/j.kint.2020.05.006. 10.1016/j.kint.2020.05.006 PMC7229463 32416116

[7] Pei G, Zhang Z, Peng J, Liu L, Zhang C, Yu C, et al. Renal involvement and early prognosis in patients with COVID-19 pneumonia. J Am Soc Nephrol. 2020. 10.1681/ASN.2020030276. 10.1681/ASN.2020030276 PMC7269350 32345702

[8] Kidney Disease: Improving Global Outcomes (KDIGO) Acute Kidney Injury Work Group. KDIGO clinical practice guideline for acute kidney injury. Kidney Int Suppl 2012; 2:1–138.

[9] Monard C, Rimmelé T, Ronco C (2019). Extracorporeal blood purification therapies for sepsis. Blood Purif.

[10] Channappanavar R, Perlman S (2017). Pathogenic human coronavirus infections: causes and consequences of cytokine storm and immunopathology. Semin Immunopathol.

[11] Ronco C, Reis T, Husain-Syed F. Management of acute kidney injury in patients with COVID-19. Lancet. 2020; 10.1016/S2213-2600(20)30229-0. 10.1016/S2213-2600(20)30229-0 PMC7255232 32416769

[12] Husain-Syed F (2016). Lung-kidney cross-talk in the critically ill patient. Am J Respir Cri Care Med.

[13] Ronco C, Reis T. Kidney involvement in COVID-19 and rationale for extracorporeal therapies. Nature Reviews Nephr. 2020; 10.1038/s41581-020-0284-7. 10.1038/s41581-020-0284-7 PMC7144544 32273593

[14] Ronco, C, Reis T, De Rosa S. Coronavirus epidemic and extracorporeal therapies in intensive care: si vis pacem para bellum. Blood Purif 2020; 10.1159/000507039 (2020). 10.1159/000507039 PMC7179535 32172242

[15] Bellomo R, Ronco C, Mehta RL, Asfar P, Boisram-Helms J. Acute kidney injury in the ICU: from injury to recovery: reports from the 5th Paris International Conference. Ann. Intensive Care 2017;7:49. DOI 10.1186/s13613-017-0260-y. 10.1186/s13613-017-0260-y PMC5418176 28474317

[16] Ronco C, Bagshaw S, Bellomo R, Clark W, Husain-Syed F, et al. Extracorporeal blood purification and organ support in the critically ill patient during COVID-19 pandemic: expert review and recommendation. Blood Purif. 2020; 10.1159/000508125. 10.1159/000508125 PMC7270067 32454500

[17] Alhazzani W, Hylander Møller M, Arabi Y, Loeb M, Ng Gong M, Honore P (2019). Cytokine removal in human septic shock: where are we and where are we going?. Ann Intensive Care.

[18] Villa G, De Rosa S, Samoni S, Neri M, Chelazzi M, Romagnoli S (2019). oXirisNet registry: a prospective, national registry on the oXiris membrane. Blood purify.

[19] Paulomi A, Marusov G, Svancara D, David J, Mor G. Simple PlexTM: a novel multi-analyte, automated microfluidic immunoassay platform for the detection of human and mouse cytokines and chemokines. Am J Reprod Immunol 2016; 75(6): 678–693. doi:10.1111/aji.12512. 10.1111/aji.12512 PMC5084752 27170460

[20] Ko M, Shum M, Lee SM et al. Performance of APACHE IV in medical intensive care unit patients: comparisons with APACHE II, SAPS 3, and MPM0 III. Acute and Critical Care 2018, 33(4):216-221DOI: 10.4266/acc.2018.00178. 10.4266/acc.2018.00178 PMC6849024 31723888

[21] Papazian L, Aubron C, Brochard L (2019). Formal guidelines: management of acute respiratory distress syndrome. Ann Intensive Care.

[22] Brzezinski M (2012). The Chen-Shapiro test for normality. Stata J.

[23] Turani F, Barchetta R, Falco M, Busatti S, Weltert L (2019). Continuous renal replacement therapy with the adsorbing filter oXiris in septic patients: a case series. Blood Purif.

[24] Zhang L, Tang G, Liu S, Cai J, Chan Y, Yang Y, Chang P (2019). Hemofilter with adsorptive capacities: case report series. Blood Purif.

[25] Broman M, Hansson F, Vincent JL, Bodelsson M. Endotoxin and cytokine reducing properties of the oXiris membrane in patients with septic shock: a randomized crossover double-blind study. PlosONE. 2019; 10.1371/journal.pone.0220444. 10.1371/journal.pone.0220444 PMC6675097 31369593

[26] Wu C, Chen X, Cai Y (2020). Risk factors associated with acute respiratory distress syndrome and death in patients with coronavirus disease 2019 pneumonia in Wuhan.

[27] Tetta C, Bellomo R, Ronco C (2003). Artificial organ treatment for multiple organ failure, acute renal failure, and sepsis: recent new trends. Artif Organs.

[28] Fu B, Xu X, Wei H (2020). Why tocilizumab could be an effective treatment for severe COVID-19?. J Transl Med.

[29] Nishimoto N, Terao K, Mima T, Nakahara H, Takagi N, Kakehi T (2008). Mechanisms and pathologic significances in increase in serum interleukin-6 (IL-6) and soluble IL-6 receptor after administration of an anti-IL-6 receptor antibody, tocilizumab, in patients with rheumatoid arthritis and Castleman disease. Blood.

[30] Sise M, BagetteM, Shepard J, Stevens J, Rhee E. Case 17-2020: a 68-year old man with COVID-19 and acute kidney injury. NEJM 2020;382(22):2147–2156. doi: 10.1056/NEJMcpc2002418. 10.1056/NEJMcpc2002418 PMC7959270 32402156

[31] Sheppard M, Laskou F, Stapleton P, Hadavi S, Dasgupta B (2017). Tocilizumab (Actemra). Hum Vaccin Immunother.

