# Supplementary material for: Blood purification therapy with a hemodiafilter featuring enhanced adsorptive properties for cytokine removal in patients presenting COVID-19: a pilot study
Source: Crit Care. 2020 Oct 12;24:605. doi: 10.1186/s13054-020-03322-6 (PMC7549343; doi:10.1186/s13054-020-03322-6)
Supplement: Supplementary file 1 — Additional file 1: Table S1. Main clinical parameters at monitoring time points 12, 24, 48 and 72 hours for control group. No patient had signs of severe systemic inflammation; only one had AKI (KDIGO stage 1); all of them had acute respiratory failure and were treated with invasive mechanical ventilation at the ICU admission. In this cohort, every patient was treated with hydroxychloroquine and azitromycine; two of them underwent treatement with lopinavir/ritornavir, while none with tocilizumab. Data are expressed as percentage N (%), mean ± standard deviation (mean±SD) and median (interquartile range, IQR) as appropriate. CKD: chronic kidney disease; CLD: chronic liver disease; CRP: C-reactive protein; INR: International Normalized Ratio; MV: mechanical ventilation; OTI: Oro-Tracheal intubation; PCT: procalcitonin; PIP: Peak inspiratory pressure; Pplat: Plateau pressure; RASS: Richmond Agitation-Sedation Score; RR: Respiratory rate; VT: tidal volume; WBC: white blood cells. [file 13054_2020_3322_MOESM1_ESM.docx]

**SUPPLEMENTARY MATERIAL**

**Table S1.** **Main clinical parameters at monitoring time points 12, 24, 48 and 72 hours for control group.** No patient had signs of severe systemic inflammation; only one had AKI (KDIGO stage 1); all of them had acute respiratory failure and were treated with invasive mechanical ventilation at the ICU admission. In this cohort, every patient was treated with hydroxychloroquine and azitromycine; two of them underwent treatement with lopinavir/ritornavir, while none with tocilizumab.

Data are expressed as percentage N (%), mean ± standard deviation (mean±SD) and median (interquartile range, IQR) as appropriate.

CKD: chronic kidney disease; CLD: chronic liver disease; CRP: C-reactive protein; INR: International Normalized Ratio; MV: mechanical ventilation; OTI: Oro-Tracheal intubation; PCT: procalcitonin; PIP: Peak inspiratory pressure; P_plat_: Plateau pressure; RASS: Richmond Agitation-Sedation Score; RR: Respiratory rate; V_T_: tidal volume; WBC: white blood cells.

|  | **12 hrs** | **24 hrs** | **48 hrs** | **72 hrs** |
| --- | --- | --- | --- | --- |
| **Clinical parameters upon RRT initiation** |  |  |  |  |
| GCS | 3 (16 100%) | 3 (16 100%) | 3 (16 100%) | 3 (14 87.5%) |
|  |  |  |  | 6 (2 12.5%) |
| Heart rate (bpm) | 95±20 | 85±15 | 85±23 | 82±8 |
| Rhythm |  |  |  |  |
| rhythmic | 16 (100%) | 16 (100%) | 16 (100%) | 16 (100%) |
| arrhythmic | 0 | 0 | 0 | 0 |
| Systolic pressure (mmHg) | 118 (24) | 106 (19) | 133 (26) | 113 (12) |
| Diastolic pressure (mmHg) | 67±12 | 66±13 | 69 (10) | 65 (6) |
| Mean pressure (mmHg) | 83 (12) | 80 (13) | 90 (11) | 80 (7) |
| Vasoactive drugs | 2 (%) | 4 (%) | 6 (%) | 4 (%) |
| Adrenaline | 2 (%) | 2 (%) | 2 (%) | 2 (%) |
| Noradrenaline | 0 (0%) | 2 (%) | 2 (%) | 0 (0%) |
| Vasopressin | 0 (0%) | 2 (%) | 2 (%) | 0 (0%) |
| Dobutamine | 0 (0%) | 0 (0%) | 2 (%) | 2 (%) |
| Dopamine | 0 (0%) | 0 (0%) | 0 (0%) | 0 (0%) |
| Vasoactive Inotropic Score (VIS) | 0 (0) | 0 (2.6) | 0 (1.4) | 0 (0.8) |
| V_T_ settings (ml) | 455±72 | 404±69 | 414±80 | 421±77 |
| RR (breaths/min) | 20±3 | 23±4 | 21±4 | 24±4 |
| PEEP (cmH2O) | 13±2 | 13±3 | 12±3 | 13±2.5 |
| PIP (cmH_2_O) | 32±8 | 30±8 | 29±13 | 34±8 |
| Mean airway pressure (cmH2O) | 29 (6) | 28 (4) | 26 (3) | 27 (3) |
| P_Plat_ (cmH2O) | 28±4 | 28±5 | 26±3 | 26±5 |
| Compliance | 32±17 | 35±15 | 38±10 | 38±15 |
| FiO2 (%) | 80 (25) | 80 (16) | 63 (13) | 55 (16) |
| PaO2 (mmHg) | 117±73 | 121±41 | 105±19 | 86±16 |
| PaO2/FiO2 | 165±120 | 163±75 | 164±37 | 150±50 |
| SaO2 (%) | 96 (7) | 98 (2) | 97 (2) | 96 (2) |
| PaCO2 (mmHg) | 50±17 | 46±7 | 47±8 | 46±5 |
| A-a O2 gradient | 363±158 | 378±114 | 207±92 | 297±127 |
| pH | 7.32±0.15 | 7.36±0.05 | 7.39±0.07 | 7.40±0.03 |
| Lactates (mmol/L) | 1.6 (1.2) | 1. 7 (0.5) | 1.8 (0.9) | 1.5 (0.7) |
| Sodium (mmol/L) | 139 (2) | 141 (3) | 143 (3) | 142 (4) |
| Potassium (mmol/L) | 4.2±1.3 | 3.9±0.7 | 4.0±0.6 | 3.9±0.6 |
| Magnesium (mg/dL) | 2.5±0.4 | 2.4±0.5 | 2.1±0.5 | 1.9±0.4 |
| Phosphate (mg/dL) | 3.3± 1.3 | 3.4±1.1 | 3.2±1.2 | 3.4±0.9 |
| Bicarbonate (mEq/L) | 22.8± 3.6 | 25.2±2.1 | 26.6±2.8 | 28.8±2.1 |
| Hematocrit (%) | 38±3 | 37.2±3.4 | 36.4±3.9 | 34.9±3.3 |
| Current creatinine (mg/dL) | 0.9 (0.2) | 1.0 (0.2) | 0.9 (0.3) | 1.1 (0.6) |
| Urinary output (ml/h) | 50 (63) | 40 (21) | 50 (12) | 50 (25) |
| 24h urinary output | 975 (950) | 900 (270) | 1340 (440) | 1356 (350) |
| Urea (mg/dL) | 58 (36) | 82 (33) | 75 (44) | 105 (27) |
| Bilirubin (mg/dl) | 0.7 (0.8) | 0.9 (0.5) | 0.7 (0.6) | 0.7 (0.9) |
| Albumin (g/dl) | 2.8 (0.2) | 2.8 (0.3) | 2.9 (0.6) | 2.9 (0.3) |
| Platelets (10^3 / μl) | 226 (66) | 236 (97) | 227 (79) | 222 (81) |
| INR | 1.2 (0.3) | 1.2 (0.2) | 1.3 (0.2) | 1.1 (0.2) |
| Antithrombin (%) | 70±25 | 69±24 | 75±18 | 82±3 |
| Fibrinogen (mg/dl) | 312 (283) | 303 (348) | 303 (360) | 586 (295) |
| D-dimers (ng/ml) | 819 (1395) | 1500 (3009) | 2280 (1004) | 2880 (1016) |
| PCT (ng/ml) | 1.14 (5) | 1.45 (0.54) | 1.43 (0.44) | 0.7 (0.6) |
| Temperature (°C) | 36.5±0.6 | 36.2±0.9 | 36.6±0.4 | 36±0.9 |
| CRP (mg/L) | 99 (154) | 288 (170) | 112 (134) | 66 (107) |
| WBC (10^3 /μl) | 11.5 (5.5) | 10.9 (1.6) | 11.6 (2.6) | 10.9 (2.8) |
| Ferritin (ng/ml) | 390±231 | 452±754 | 797±950 | 1180±900 |
| IL-6 (ng/l) | 208 (135) | 460 (88) | 350 (255) | 415 (144) |
| Total SOFA Score | 8.5 (2.5) | 8 (2) | 7.5 (2) | 8 (2) |
